# Person-related work and the risk of cardiovascular disease: a Swedish register-based cohort study

**DOI:** 10.1093/eurpub/ckaf080

**Published:** 2025-05-28

**Authors:** Kuan-Yu Pan, Melody Almroth, Alicia Nevriana, Tomas Hemmingsson, Katarina Kjellberg, Daniel Falkstedt

**Affiliations:** Unit of Occupational Medicine, Institute of Environmental Medicine, Karolinska Institutet, Stockholm, Sweden; Unit of Occupational Medicine, Institute of Environmental Medicine, Karolinska Institutet, Stockholm, Sweden; Unit of Integrative Epidemiology, Institute of Environmental Medicine, Karolinska Institutet, Stockholm, Sweden; Unit of Occupational Medicine, Institute of Environmental Medicine, Karolinska Institutet, Stockholm, Sweden; Department of Public Health Sciences, Stockholm University, Stockholm, Sweden; Unit of Occupational Medicine, Institute of Environmental Medicine, Karolinska Institutet, Stockholm, Sweden; Centre for Occupational and Environmental Medicine, Region Stockholm, Stockholm, Sweden; Unit of Occupational Medicine, Institute of Environmental Medicine, Karolinska Institutet, Stockholm, Sweden

## Abstract

Person-related work requires interaction with individuals not employed at the workplace, such as clients and patients, and can result in emotional labour, emotional demands, and confrontation. These stressors may increase workers’ risk of cardiovascular disease (CVD), including coronary heart disease (CHD) and stroke, whereas colleagues’ support may help buffer their impact. We aimed to examine the association between person-related work and the risk of CVD, and effect modification of social support at work. The study included around two million CVD-free workers aged 40–60 years in Sweden in 2006. Three dimensions of person-related work, including general contact with people, emotional demands, and confrontation, and job control and social support were respectively assessed using job exposure matrices. CVDs in 2007–20 were recorded in patient and death registers. Multivariable Cox regression models were used. A total of 114 404 individuals developed CVD (65 857 CHD and 48 547 stroke). High exposures to the three dimensions were associated with 4%–12% increased risks of CVD (7%–20% for CHD and 2%–7% for stroke) in women and 2%–8% (2%–7% for CHD and 3%–10% for stroke) in men. Adjusting for job control attenuated the associations for general contact with people in women. The increased risks related to emotional demands and confrontation in women and general contact with people and confrontation in men were not present in those more likely to receive high social support. In conclusion, person-related work is associated with an increased risk of CVD, and social support at work seems to modify the magnitude of this association.

## Introduction

Cardiovascular disease (CVD) poses a great burden to societies worldwide [1]. Although CVD mortality has been declining in high-income countries, such as Sweden [2], it remains the number one cause of death for both men and women [1]. There has been a growing body of evidence regarding work-related psychosocial factors, such as work stress [3–6], and the long-term risk of CVD, including coronary heart disease (CHD) and stroke. However, the potential effect of exposures within person-related work on CVD has not been studied.

Person-related work requires face-to-face or voice-to-voice interaction with individuals who are not employed at the workplace. Examples are patients, customers, clients, passengers, or students [7].

Several dimensions of person-related work may cause stress which can in turn increase the risk of CVD [8]. Firstly, a high level of contact with people who are not employed at the workplace means that workers need to constantly manage their emotions in order to express organizationally expected emotions. This emotional labour can be especially burdensome when there is dissonance between displayed emotions and genuinely felt emotions [7]. Secondly, emotional demands are high in some person-related work, such as human service occupations, including healthcare, social care, and education [9]. Working in these occupations requires empathy and emotional involvement with the clients, who may be in difficult, painful, or distressing situations [10]. Thirdly, workers in person-related work deal with customers’ needs and problems which can sometimes lead to confrontation [11].

In addition to pathophysiological alterations related to stress response, stressors in person-related work may also result in burnout [12] and behavioural changes, such as over-eating [13], excessive alcohol consumption [14], and sleep problems [15]. These behavioural changes can contribute to the risk of CVD in person-related work as well [16].

The dimensions of person-related work that can distress workers, such as emotional demands, may in part represent the nature of such occupations and thus may not be completely avoidable. It is therefore crucially important to identify resources at the workplace that may help attenuate the distress caused by these situations. One of the candidates is social support provided by supervisors and colleagues [17]. Indeed, it has been shown that vertical and horizontal psychosocial resources, including support received from supervisors and peers, are associated with a lower risk of CVD [18]. It is plausible that the risk of CVD in person-related work may differ depending on the level of social support perceived at the workplace.

In this study, for the first time, we aimed to investigate the prospective association between three dimensions of person-related work—general contact with people, emotional demands, and confrontation—and the risk of CVD, and to examine whether social support at work modifies the association. We hypothesized that higher exposures to the three dimensions are respectively associated with a higher risk of CVD, and that the increased risk is attenuated by higher social support at work.

## Methods

### Study population

This study was based on the Swedish Work, Illness, and labour-market Participation cohort which includes all (around 5.4 million) individuals who were aged between 16 and 65 years and registered in Sweden in 2005. Data were retrieved from several Swedish administrative and medical registers, including the total population register [19], the longitudinal integrated database for health insurance and labour market studies (LISA) register [20], the national in- and out-patient registers [21], the cause of death register, the prescribed drug register [22], as well as information from earlier population censuses. Linkages between registers were made by Statistics Sweden using personal identification numbers.

The present study included CVD-free individuals who were aged 40–60 years and had information on job held in 2006 (baseline). The selection of baseline year was mainly due to the use of the prescribed drug register, which did not cover the whole Swedish population until late 2005, for important confounding control [22]. This resulted in a final population of 2 064 766, of which 50.9% were women.

Ethical approval was obtained by the Regional Ethics Review Board in Stockholm reference number 2017/1224-31, 2018/1675-32, and 2022/02725-02.

### Exposure

Occupational information is available in the LISA register administered by Statistics Sweden, coded based on the Swedish ISCO-88 four-digit classification of occupations. We retrieved the occupational code in 2006 for each individual.

To evaluate the three dimensions of person-related work, we constructed a job exposure matrix (JEM) based on the Swedish Work Environment Surveys (1997–2013). Questions and answer options are shown in Table 1. For around 350 occupations we calculated the proportion of responses to a certain level of exposure, separately for women and men. For the ‘general contact with people’ dimension, similar to a previous study [23], we calculated the proportion of responses to ‘roughly three-fourths of the time’ or ‘almost all the time’. For ‘emotional demands’ and ‘confrontation’ dimensions, we calculated the proportion of responses to ‘a few days per week” or ‘every day’.

**Table 1.** Questions and answer options in the Swedish Work Environment Surveys (1997-2013) by dimensions of person-related work

Dimension	Question	Answer option
General contact with people	Do you have anything to do with people at work who are not employed at the workplace (e.g. patients, customers, clients, passengers, students, etc.)?	Not at all, roughly 1/10 of the time, roughly ¼ of the time, half of the time, roughly ¾ of the time, almost all the time
Emotional demands	Does it happen that through work you come into close contact with the seriously ill or people with serious problems?	Not at all/rarely the last 3 months, a few days per month, one day per week, a few days per week, every day
Confrontation	Are you involved in any form of conflict or quarrel in your workplace with other people (e.g. patients, customers, clients, passengers, students)?	Not at all the last 12 months, sometime the last 12 months, a few times the last 3 months, a few days per month, one day per week, a few days per week, every day

For occupations that had fewer than 10 respondents in the surveys, we imputed exposures in these occupations using exposures in similar occupations that had 10 or more respondents. A similar approach has been used for constructing a JEM for job control, job demands, and social support at work using the Swedish Work Environment Surveys [24].

We linked the JEM to the study population using their occupational codes in 2006. We categorized each dimension into three levels based on the tertile distribution in the study population. Spearman correlations between the three dimensions were moderate, ranging between 0.48 and 0.51 (Supplementary Table S1).

In both women and men, the occupations with the highest level of exposure to each dimension encompassed sectors of healthcare, education, service industry, hospitality, social work, legal professional, guards, and transportation (Supplementary Tables S2–S4).

### Outcome

Incident CVD included the first diagnosis of CHD or stroke between 2007 and 2020. In line with previous studies [3, 4], CHD included myocardial infarction (ICD-10 codes I21–I22) from the national patient registers and coronary deaths (ICD-10 codes I20–I25) from the cause of death register. Stroke (ICD-10 code I61, I63–I64) was extracted from the national patient registers and the cause of death register.

### Covariates

As potential confounders, we included age, birth country, education, civil status, early-life socioeconomic position (SEP), and mental and cardiometabolic health conditions, as these factors have been associated with both occupational choices and CVD [25, 26]. Low job control has been found to be present in some person-related work and has been associated with CVD as well [3, 5, 6, 9]. We adjusted for job control to estimate the independent impact of the three dimensions of person-related work on CVD. We considered social support at work as an effect modifier.

Information on age, birth country, education, and civil status was obtained from the LISA register. Birth country was categorized according to whether the individual was born in Sweden or not. Highest attained education was categorized as compulsory school only (≤9 years), vocational (10–11 years), upper secondary (12 years), post-secondary (13–14 years), and university (≥15 years). Civil status was categorized as married/partnered, unmarried, divorced, and widowed.

For mental health condition, history of psychiatric diagnosis (ICD-10 codes F01-F99 and ICD-9/8 29-31) was obtained from the national patient registers in or before 2006. For cardiometabolic health condition, history of medication use for treating diabetes (ATC code A10), dyslipidaemia or hypertension (ATC codes C02-C10) was obtained from the prescribed drug register in 2005 and 2006.

Individuals were linked to their parents; we used information from the population and housing censuses from 1960 (for those born 1941–54), 1970 (for those born 1955–64), 1980 (for those born 1965–74) and 1990 (for those born 1975–89) to capture their early-life SEP. This was estimated according to the father’s occupation, or mother’s occupation if father’s was missing, and categorized as non-manual employees at a higher level, non-manual employees at an intermediate level, assistant non-manual employees, skilled manual workers, non-skilled manual workers, farmers, and those with no parental occupation documented.

Job control and social support at work in 2006 were assessed using JEMs based on the Swedish Work Environment Surveys (1997–2013). Questions in the surveys are shown in Supplementary Table S5. They were dichotomized using the medians in the study population, respectively [24].

### Statistical analysis

Baseline characteristics of the study population were explored according to the outcome by the end of follow-up period, as well as the levels of dimensions of person-related work.

The incidence rate of CVD was estimated for each level of the three person-related work variables. Cox proportional hazard regression models with age as the underlying timescale were built to estimate hazard ratios (HR) and 95% confidence intervals (CI) for associations of the person-related work variables with risk of CVD. Person-time was counted from 1 January 2007 until the incidence of CVD, death, emigration, or the end of the follow-up period on 31 December 2020, whichever came first.

Effect modification of social support at work was tested by entering an interaction term of the person-related work variable and social support in the model. For this analysis the medium level of the person-related work variable was omitted for simplification of interpretation. We used likelihood ratio test (LRT) to test the overall significance of the interaction. Interaction that had *P* < .05 was considered as modifying the association between the person-related work variable and CVD [27]. To further interpret the interaction, we estimated associations of combinations of the person-related work variables and social support with CVD.

Additional analyses were performed looking at CHD and stroke as separate outcomes.

Women and men tend to hold different occupations and positions and may have different types of exposures even within the same occupations. It is especially important for person-related work because many of the occupations are female-dominated. Furthermore, the general risk of CVD differs between women and men [28]. Therefore, all analyses were done for women and men separately.

Model 1 was adjusted for age, birth year, civil status, birth country, early-life SEP, education, mental and cardiometabolic health conditions. Model 2 was additionally adjusted for job control.

Data management and statistical analyses were done using STATA 17 (StataCorp LLC, College Station, TX, USA)

## Results

From 2007 to 2020 (over 27 626 825.5 person-years), 114 404 individuals (68.9% men) developed CVD, including 65 857 CHD cases and 48 547 stroke cases. In both women and men, those who developed CVD, compared to those who did not, were older and more likely to have lower education, a history of psychiatric diagnosis, medication use for hypertension, dyslipidaemia or diabetes, and low job control. Their parents were less likely to be non-manual workers (Table 2).

**Table 2.** Baseline characteristics according to incidence of CVD by sex

	Men (*N* = 1 011 775)		Women (*N* = 1 052 991)	
CVD	Yes *N* = 78 772	No *N* = 933 003	Yes *N* = 35 632	No *N* = 1 017 359
Characteristics	%	%	%	%
Age				
40–49	30.4	52.0	27.7	49.8
50–60	69.6	48.0	72.3	50.2
Foreign born	13.1	12.1	12.9	13.1
Education years				
≤9	24.5	18.3	18.8	12.0
10–11	35.3	35.0	42.0	35.5
12	14.3	13.7	11.1	13.4
13–14	12.2	14.8	13.3	17.6
≥15	13.7	18.2	14.8	21.5
Civil status				
Married/partnered	54.0	55.8	53.7	57.5
Unmarried	27.5	29.6	20.6	22.8
Divorced	17.6	14.0	22.6	18.0
Widowed	0.9	0.6	3.1	1.7
Parents’ occupation				
Non-manual higher level	4.0	5.5	3.7	5.2
Non-manual intermediate level	13.4	16.5	12.6	15.8
Non-manual assistant	9.4	10.5	9.1	10.3
Skilled manual	24.3	23.7	24.9	23.6
Non-skilled manual	26.3	26.2	27.3	23.6
Farmer	7.0	7.0	6.5	6.6
No record	15.6	15.6	15.9	14.9
History of psychiatric diagnosis	5.3	3.7	6.7	4.2
History of medication use for hypertension or dyslipidaemia	29.3	16.7	35.5	18.1
History of medication use for diabetes	7.3	2.9	6.8	1.8
Low job control	57.7	51.1	57.5	49.9
Low social support at work	50.8	50.1	49.7	51.9

Low job control was associated with a higher risk of CVD, CHD, and stroke, respectively, in both women and men, while low social support at work was associated with these outcomes in women, but not in men (Supplementary Table S6).

Men who had high exposures to the three dimensions of person-related work were more likely to have higher education and a history of psychiatric diagnosis, medication use for hypertension, dyslipidaemia or diabetes, and their parents were more likely to be non-manual workers. Men who had high emotional demands and confrontation were older and more likely to be foreign born. In women, those who had high exposures to the three dimensions of person-related work were more likely to have higher education. Women who had high exposures to general contact with people and emotional demands were more likely to have low job control, while those with high exposures to confrontation were less likely to have low job control. Finally, women who had high emotional demands and confrontation were more likely to have a history of psychiatric diagnosis and low social support at work (Supplementary Tables S7–S9).

Across dimensions of person-related work, incidence rates for CVD, CHD and stroke in men (5.45–6.17, 3.42–3.83, 2.04–2.35 per 1000 person-years, respectively) were higher than those in women (2.31–2.71, 1.07–1.34, 1.24–1.38 per 1000 person-years, respectively). In both women and men, high exposures to the three dimensions, compared to low exposures, were associated with a modestly (4%–12% in women and 2%–8% in men) increased risk of CVD in Model 1. Further adjusting for job control slightly attenuated some effect estimates but most associations remained statistically significant. The only exception was the association between a high level of general contact with people and the risk of CVD in women (HR 0.99; 95% CI: 0.96–1.02). A similar pattern was observed for both CHD and stroke (Table 3).

**Table 3.** Number of cases, incidence rates (95% CI), and HRs (95% CI) for CVD, CHD and stroke by dimensions of person-related work

	CVD				CHD				Stroke			
	*N* cases	Incidence rate per 1000 person-years (95% CI)	Model 1 HR (95% CI)	Model 2 HR (95% CI)	N cases	Incidence rate per 1000 person-years (95% CI)	Model 1 HR (95% CI)	Model 2 HR (95% CI)	N cases	Incidence rate per 1000 person-years (95% CI)	Model 1 HR (95% CI)	Model 2 HR (95% CI)
Women												
General contact with people												
Low	12 223	2.51 (2.46–2.55)	Ref	Ref	5808	1.19 (1.16–1.22)	Ref	Ref	6415	1.32 (1.28–1.35)	Ref	Ref
Medium	12 240	2.65 (2.60–2.69)	1.07 (1.05–1.10)	1.06 (1.03–1.08)	5851	1.27 (1.23–1.30)	1.09 (1.05–1.13)	1.06 (1.02–1.10)	6389	1.38 (1.35–1.42)	1.06 (1.03–1.10)	1.05 (1.02–1.09)
High	11 169	2.34 (2.30–2.39)	1.04 (1.01–1.07)	0.99 (0.96–1.02)	5284	1.11 (1.08–1.14)	1.07 (1.03–1.11)	1.00 (0.96–1.04)	5885	1.24 (1.20–1.27)	1.02 (0.98–1.06)	0.98 (0.95–1.02)
Emotional demands												
Low	11 817	2.48 (2.44–2.53)	Ref	Ref	5551	1.17 (1.14–1.20)	Ref	Ref	6266	1.32 (1.28–1.35)	Ref	Ref
Medium	11 256	2.31 (2.27–2.35)	0.99 (0.96–1.01)	0.97 (0.94–1.00)	5198	1.07 (1.04–1.10)	0.99 (0.96–1.03)	0.97 (0.93–1.01)	6058	1.24 (1.21–1.27)	0.98 (0.95–1.02)	0.97 (0.94–1.01)
High	12 559	2.71 (2.67–2.76)	1.12 (1.09–1.15)	1.08 (1.06–1.11)	6194	1.34 (1.31–1.37)	1.20 (1.16–1.25)	1.15 (1.10–1.19)	6365	1.38 (1.34–1.41)	1.06 (1.02–1.09)	1.04 (1.00–1.08)
Confrontation												
Low	11 772	2.41 (2.36–2.45)	Ref	Ref	5510	1.13 (1.10–1.16)	Ref	Ref	6262	1.28 (1.25–1.31)	Ref	Ref
Medium	11 906	2.50 (2.45–2.54)	1.00 (0.98–1.03)	0.99 (0.97–1.02)	5753	1.21 (1.18–1.24)	1.04 (1.00–1.08)	1.02 (0.98–1.06)	6153	1.29 (1.26–1.32)	0.98 (0.94–1.01)	0.97 (0.93–1.01)
High	11 954	2.60 (2.55–2.64)	1.09 (1.06–1.12)	1.09 (1.06–1.12)	5680	1.23 (1.20–1.27)	1.12 (1.08–1.16)	1.11 (1.07–1.16)	6274	1.36 (1.33–1.40)	1.07 (1.03–1.11)	1.07 (1.03–1.10)
Men												
General contact with people												
Low	27 058	5.92 (5.85–5.99)	Ref	Ref	16 834	3.69 (3.63–3.74)	Ref	Ref	10 224	2.24 (2.20–2.28)	Ref	Ref
Medium	26 838	5.79 (5.72–5.86)	0.97 (0.95–0.99)	0.99 (0.97–1.00)	16 716	3.61 (3.55–3.66)	0.98 (0.96–1.01)	1.00 (0.97–1.02)	10 122	2.18 (2.14–2.23)	0.96 (0.93–0.98)	0.97 (0.95–1.00)
High	24 876	5.99 (5.92–6.07)	1.04 (1.02–1.06)	1.04 (1.02–1.06)	15 364	3.70 (3.64–3.76)	1.04 (1.01–1.06)	1.04 (1.01–1.06)	9512	2.29 (2.25–2.34)	1.04 (1.01–1.07)	1.04 (1.01–1.07)
Emotional demands												
Low	24 933	5.45 (5.39–5.52)	Ref	Ref	15 620	3.42 (3.36–3.47)	Ref	Ref	9318	2.04 (2.00–2.08)	Ref	Ref
Medium	26 933	6.08 (6.01–6.15)	1.06 (1.04–1.08)	1.05 (1.03–1.07)	16 629	3.75 (3.70–3.81)	1.04 (1.02–1.06)	1.03 (1.01–1.05)	10 310	2.33 (2.28–2.37)	1.09 (1.06–1.12)	1.08 (1.05–1.11)
High	26 906	6.17 (6.10–6.25)	1.08 (1.06–1.10)	1.05 (1.03–1.07)	16 684	3.83 (3.77–3.89)	1.07 (1.05–1.10)	1.03 (1.01–1.06)	10 230	2.35 (2.30–2.39)	1.10 (1.07–1.13)	1.07 (1.03–1.10)
Confrontation												
Low	26 448	5.89 (5.82–5.96)	Ref	Ref	16 509	3.68 (3.62–3.73)	Ref	Ref	9943	2.21 (2.17–2.26)	Ref	Ref
Medium	26 397	5.90 (5.83–5.98)	1.00 (0.99–1.02)	1.03 (1.01–1.04)	16 432	3.67 (3.62–3.73)	1.01 (0.98–1.03)	1.03 (1.00–1.05)	9970	2.23 (2.19–2.27)	1.01 (0.98–1.03)	1.03 (0.99–1.06)
High	25 927	5.90 (5.82–5.97)	1.02 (1.01–1.04)	1.03 (1.01–1.05)	15 992	3.63 (3.58–3.69)	1.02 (1.01–1.05)	1.02 (1.00–1.04)	9945	2.26 (2.22–2.31)	1.03 (1.01–1.06)	1.04 (1.01–1.07)

Model 1 adjusting for age, birth year, civil status, birth country, early-life SEP, education, mental and cardiometabolic health conditions.

Model 2 adjusting for age, birth year, civil status, birth country, early-life SEP, education, mental and cardiometabolic health conditions, and job control.

There was a sign of effect modification by social support at work, although the LRTs were not always statistically significant. The associations between high exposures to the three dimensions of person-related work and the risk of CVD were higher among individuals with low social support than those with high social support in both women and men. Again, the only exception was general contact with people in women since it was not associated with CVD after adjusting for job control. Specifically, the risk estimates of CVD in relation to high exposures to emotional demands and confrontation were no longer statistically significant among those with high social support in women. This was also true regarding general contact with people and confrontation in men. Again, there was a similar pattern when looking at CHD and stroke separately as the outcome (Table 4).

**Table 4.** Incidence rates (95% CI) and HRs (95% CI) for CVD, CHD and stroke by combinations of dimensions of person-related work and social support at work

		CVD			CHD			Stroke		
		Incidence rate per 1000 person-years (95% CI)	Model 2 HR (95% CI)	*P*-value of likelihood-ratio test	Incidence rate per 1000 person-years (95% CI)	Model 2 HR (95% CI)	*P*-value of likelihood-ratio test	Incidence rate per 1000 person-years (95% CI)	Model 2 HR (95% CI)	*P*-value of likelihood-ratio test
**Women**										
**General contact with people**	**Social support at work**			0.16			0.60			0.16
Low	High	2.53 (2.48–2.59)	Ref		1.21 (1.17–1.25)	Ref		1.33 (1.29–1.37)	Ref	
Low	Low	2.46 (2.39–2.54)	1.04 (0.99–1.08)		1.16 (1.12–1.21)	1.02 (0.97–1.08)		1.30 (1.25–1.35)	1.05 (0.99–1.11)	
High	High	2.72 (2.65–2.79)	1.01 (0.97–1.05)		1.35 (1.30–1.40)	1.02 (0.97–1.08)		1.37 (1.32–1.42)	1.00 (0.95–1.06)	
High	Low	2.02 (1.96–2.07)	0.97 (0.93–1.02)		0.90 (0.86–0.94)	0.98 (0.91–1.04)		1.12 (1.08–1.16)	0.97 (0.91–1.03)	
**Emotional demands**	**Social support at work**			<0.001			<0.001			<0.001
Low	High	2.70 (2.65–2.76)	Ref		1.29 (1.25–1.33)	Ref		1.41 (1.37–1.45)	Ref	
Low	Low	1.99 (1.92–2.06)	0.99 (0.94–1.03)		0.89 (0.84–0.94)	0.97 (0.91–1.04)		1.10 (1.05–1.16)	0.99 (0.94–1.05)	
High	High	2.77 (2.69–2.86)	1.00 (0.97–1.04)		1.41 (1.35–1.47)	1.05 (0.99–1.11)		1.37 (1.31–1.43)	0.96 (0.91–1.02)	
High	Low	2.68 (2.63–2.74)	1.14 (1.09–1.19)		1.30 (1.27–1.35)	1.22 (1.15–1.31)		1.38 (1.34–1.42)	1.08 (1.02–1.15)	
**Confrontation**	**Social support at work**			<0.001			<0.001			<0.01
Low	High	2.51 (2.45–2.57)	Ref		1.19 (1.15–1.23)	Ref		1.32 (1.28–1.37)	Ref	
Low	Low	2.29 (2.23–2.36)	1.05 (1.01–1.09)		1.06 (1.02–1.10)	1.04 (0.98–1.10)		1.24 (1.19–1.28)	1.06 (1.01–1.11)	
High	High	2.63 (2.53–2.73)	0.97 (0.93–1.02)		1.22 (1.15–1.29)	0.93 (0.87–1.00)		1.41 (1.34–1.49)	1.02 (0.95–1.08)	
High	Low	2.59 (2.54–2.64)	1.10 (1.07–1.14)		1.24 (1.20–1.28)	1.16 (1.11–1.22)		1.35 (1.31–1.39)	1.05 (1.00–1.10)	
**Men**										
**General contact with people**	**Social support at work**			<0.001			<0.05			<0.01
Low	High	5.99 (5.90–6.07)	Ref		3.72 (3.65–3.79)	Ref		2.27 (2.22–2.32)	Ref	
Low	Low	5.79 (5.66–5.91)	1.02 (0.99–1.04)		3.61 (3.52–3.71)	1.02 (0.99–1.06)		2.17 (2.10–2.25)	1.01 (0.96–1.05)	
High	High	5.73 (5.62–5.84)	1.01 (0.99–1.04)		3.57 (3.49–3.66)	1.02 (0.99–1.05)		2.16 (2.09–2.23)	1.00 (0.96–1.04)	
High	Low	6.21 (6.11–6.32)	1.05 (1.02–1.08)		3.81 (3.73–3.89)	1.04 (1.01–1.08)		2.40 (2.34–2.47)	1.07 (1.02–1.11)	
**Emotional demands**	**Social support at work**			0.30			0.86			0.06
Low	High	5.26 (5.17–5.35)	Ref		3.29 (3.22–3.37)	Ref		1.97 (1.91–2.03)	Ref	
Low	Low	5.66 (5.56–5.76)	1.01 (0.98–1.03)		3.55 (3.47–3.63)	1.01 (0.98–1.04)		2.11 (2.05–2.17)	1.00 (0.96–1.04)	
High	High	6.15 (6.04–6.26)	1.04 (1.01–1.07)		3.87 (3.78–3.96)	1.04 (1.01–1.07)		2.28 (2.21–2.35)	1.04 (0.99–1.08)	
High	Low	6.19 (6.10–6.29)	1.05 (1.02–1.08)		3.80 (3.72–3.87)	1.03 (1.00–1.06)		2.40 (2.34–2.46)	1.09 (1.04–1.13)	
**Confrontation**	**Social support at work**			0.10			0.30			0.14
Low	High	5.95 (5.86–6.05)	Ref		3.72 (3.65–3.79)	Ref		2.23 (2.18–2.29)	Ref	
Low	Low	5.79 (5.68–5.91)	0.98 (0.95–1.00)		3.61 (3.52–3.70)	0.98 (0.95–1.01)		2.18 (2.12–2.25)	0.98 (0.94–1.02)	
High	High	5.94 (5.84–6.05)	1.01 (0.99–1.03)		3.70 (3.62–3.78)	1.01 (0.98–1.04)		2.24 (2.18–2.30)	1.02 (0.98–1.05)	
High	Low	5.85 (5.75–5.95)	1.05 (1.02–1.08)		3.56 (3.49–3.64)	1.03 (1.00–1.07)		2.28 (2.22–2.35)	1.07 (1.03–1.12)	

Model 2 adjusting for age, birth year, civil status, birth country, early-life SEP, education, mental and cardiometabolic health conditions, and job control.

The medium level of dimensions of person-related work was omitted in this analysis.

## Discussion

In this large register-based cohort study, we investigated the risk of CVD in relation to three dimensions of person-related work. We found that in both women and men, high levels of general contact with people, emotional demands, and confrontation are respectively associated with a moderately increased risk of CVD. However, in women the association for general contact with people was no longer statistically significant after taking job control into account. Furthermore, social support at work appeared to modify the associations. The increased risks related to emotional demands and confrontation in women, and general contact with people and confrontation in men were shown among those more likely to receive low social support, but not among those more likely to receive high social support at work.

To our knowledge, the current study is the first that studied specific dimensions of person-related work and the risk of CVD. Regarding other outcomes of interest, person-related work and emotional demands at work have both been associated with a higher risk of mental health problems [23, 29] and sickness absence [30]. In particular, the item used to refer to emotional demands in our study was the same as ‘content-related emotional demands’ in a previous study [30].

Our findings are generally in line with our hypotheses that high levels of exposures to the three dimensions of person-related work are associated with a higher risk of CVD. They support the notion that working with people in general and with people who are in problematic situations in particular, as well as conflict and confrontation occurring at the workplace, can generate stress that affects workers’ cardiovascular health [8].

Specifically, when having contact with people, there are behavioural expectations about which emotions ought to be expressed and which ought to be hidden according to societal, occupational, and organizational norms [17]. While it may be especially demanding when there is dissonance between displayed emotions and genuinely felt emotions [7], it is also argued that some degree of effort is required in expressing emotions even in situations where there is congruence between the worker’s felt emotions and the organizationally desired emotions. For example, the feeling of sympathy should generally be expressed with the appropriate facial display and tone of voice, rather than floods of tears [17].

Working with individuals who are ill or with serious problems, such as human service occupations, may be especially emotionally demanding. Healthcare professionals and social workers, for example, take responsibility for the fundamental human needs of the client and witness human suffering [31]. In the long run this can result in compassion fatigue and burnout, especially because of a lack of reciprocity in relations with clients and patients [32]. Indeed, previous studies have associated human service occupations with a higher risk of mental health problems [23] and sickness absence [9].

The finding of confrontation, including conflict and quarrel with people, associated with a higher CVD risk to some extent echoes a study that showed the impact of violence at work, mainly originated from outside the organization (e.g. clients), on cardiovascular health [33]. Person-related work, especially among social and healthcare sectors, has been shown to entail exposures to work-related threats and violence [34]. In addition, exposures to workplace violence have been suggested to explain the higher risk of sickness absence among workers in human service occupations [9].

Several work-related factors, such as low job control [3], effort-reward imbalance [35], long working hours [36], and workplace sexual harassment [37] have been associated with a higher risk of CVD. These adverse working conditions may also exist in person-related work. Indeed, previous studies showed that low job control contributed to the higher risk of sickness absence among workers in human service occupations [9]. In our study, we observed that the risks of CVD were attenuated, especially regarding general contact with people in women, after adjusting for job control. Future studies are encouraged to investigate the role of other work-related factors in the risk of CVD in person-related work.

In line with our hypotheses, we found that high social support at work appeared to buffer the impact of person-related work on CVD. Since emotional demands and confrontation may be inherently more common in person-related work, these findings suggest the importance of social support, both received from supervisors and peer colleagues, in potentially reducing stress and its impact on CVD. It is noteworthy, however, that items used to assess social support at work in the Swedish Work Environment Surveys were generic and not specific to scenarios and demands in person-related work. More research is needed to develop context-specific support systems tailored for different sectors in person-related work.

We observed a slightly different pattern between women and men regarding the association between general contact with people and CVD, where job control appeared to be more of an explanatory factor in women than in men. While occupations categorised as high in general contact with people were similar in both sexes in our data, we observed that women in this group were more likely to have low job control than were the men (Supplementary Table S7). This observation may have confirmed the sex-based segregation in hierarchical position and differences in exposure, even in the same occupation. Moreover, the associations regarding emotional demands and confrontation were stronger in women, especially for CHD. This may be explained by women’s potentially higher exposure magnitude than men’s. However, it could also be due to the higher prevalence of risk factors for CVD in men, such as smoking, hypertension, and overweight [38], which may somewhat dilute the explanatory role of work-related exposures.

This study has several strengths, including the nation-wide, representative sample which can help reduce selection or attrition bias. The use of JEMs to assess three dimensions of person-related work, as well as job control and social support at work, allows for measures less affected by other factors and reduces reporting bias. Particularly, emotional demands assessed in the study represented content-related emotional demands, which has been shown to be less biased by mental health issues compared to perceived emotional demands [30]. Instead of focusing on specific occupations, our approach of focusing on three dimensions allowed us to categorise occupations based on common characteristics regarding exposures in person-related work. Furthermore, important confounders, including mental and cardiometabolic health conditions and early-life SEP, were considered.

Some limitations deserve to be acknowledged. JEMs assess job exposures on the occupational level and do not capture the potential variations of individuals’ experiences or work environment within a particular occupation. We were also unable to assess workers’ true feelings and reactivity when dealing with people. These are likely to result in non-differential misclassification of psychosocial exposures at work, which may lead to underestimation of true associations. For each dimension of person-related work, we only used one item and thus may have missed other aspects related to the dimension. Additionally, we only measured occupational exposures at one point in time and did not consider the possible change of occupations. We have, however, found psychosocial work environment at the occupational level to be quite stable over time [39].

The patient registers started from 1973 onwards so medical histories prior to 1973 are not available. Additionally, diagnoses of disorders in the registers tend to be more severe cases including those who get hospital or specialised treatment and miss milder untreated cases or cases treated in primary care. However, we believe that for myocardial infarction and stroke it would be less of a concern. Furthermore, individuals drawn to person-related jobs may be extroverted and sociable. These personality traits have been associated with higher alcohol consumption [40], which may in turn increase the risk of CVD among these workers. Unhealthy behaviours may also be a way that workers use to cope with stress in person-related work [16]. However, detailed lifestyle factors are not available in the register-based study, and thus we were unable to disentangle biological (i.e. pathological changes due to stress response) and behavioural mechanisms underlying the associations observed.

In conclusion, person-related work is associated with an increased risk of CVD. This finding highlights the demands and challenges of interpersonal relationships in person-related work and their potential impact on workers’ risk of CVD. However, providing social support in person-related work might be beneficial in this context.

## Supplementary Material

ckaf080_Supplementary_Data

## Data Availability

Data are available on reasonable request. Data may be obtained from a third party and are not publicly available. The data that support the findings of this study are available from Statistics Sweden but restrictions apply to the availability of these data, which were used under license for the current study, and are not publicly available. Data are, however, available from the authors on reasonable request and with permission of Statistics Sweden. Key points Adverse psychosocial work environment can affect cardiovascular health. Working with people was related to a higher risk of CVD. The relationship was not present in workers more likely to receive high social support at work. Providing social support in person-related work might help reduce the risk of CVD.
