# Supplementary material for: Person-related work and the risk of cardiovascular disease: a Swedish register-based cohort study
Source: Eur J Public Health. 2025 May 28;35(4):657–64. doi: 10.1093/eurpub/ckaf080 (PMC12311333; doi:10.1093/eurpub/ckaf080)
Supplement: ckaf080_Supplementary_Data [file ckaf080_supplementary_data.docx]

**Supplemental Material**

**Table S1. Spearman correlations between the three dimensions of person-related work**

|  | General contact with people | Emotional demands | Confrontation |
| --- | --- | --- | --- |
| General contact with people | 1 |  |  |
| Emotional demands | 0.51 | 1 |  |
| Confrontation | 0.48 | 0.49 | 1 |

**Table S2. Twenty occupations with the highest exposure to general contact with people by sex**

| **Men** | |
| --- | --- |
| Occupational code | Occupational title |
| 3234 | Psychiatric nurses |
| 2235 | District nurses |
| 2234 | Paediatric nurses |
| 3225 | Dental hygienists |
| 2222 | Dentists |
| 3239 | Nursing associate professionals not elsewhere classified |
| 2233 | Emergency room nurses |
| 2231 | Midwives |
| 3226 | Physiotherapists and related associate professionals |
| 3221 | Occupational therapists |
| 3232 | Surgery nurses |
| 3233 | Geriatric nurses |
| 3231 | Nursing associate professionals |
| 3223 | Dieticians |
| 3224 | Optometrists and opticians |
| 3228 | Pharmaceutical assistants |
| 3229 | Other therapists |
| 4221 | Travel agency and related clerks |
| 5111 | Travel attendants and travel stewards |
| 5226 | Car, boat and caravan salesmen |
| **Women** | |
| 5112 | Transport conductors |
| 2222 | Dentists |
| 8322 | Bus and tram drivers |
| 2233 | Emergency room nurses |
| 3225 | Dental hygienists |
| 3224 | Optometrists and opticians |
| 5111 | Travel attendants and travel stewards |
| 3417 | Appraisers, valuers and auctioneers |
| 2235 | District nurses |
| 2231 | Midwives |
| 5135 | Dental nurses |
| 3229 | Other therapists |
| 3228 | Pharmaceutical assistants |
| 5225 | Gas station managers |
| 2225 | Speech therapists |
| 2229 | Health care professionals not elsewhere classified |
| 2460 | Religious professionals |
| 8311 | Train drivers |
| 5142 | Undertakers |
| 8321 | Car and taxi drivers |

**Table S3. Twenty occupations with the highest exposure to emotional demands by sex**

| **Men** | |
| --- | --- |
| Occupational code | Occupational title |
| 3233 | Geriatric nurses |
| 3232 | Surgery nurses |
| 3231 | Nursing associate professionals |
| 2233 | Emergency room nurses |
| 2231 | Midwives |
| 5153 | Prison guards |
| 5132 | Assistant nurses and hospital ward assistants |
| 3234 | Psychiatric nurses |
| 2221 | Medical doctors |
| 2235 | District nurses |
| 2234 | Paediatric nurses |
| 3461 | Social workers and related associate professionals |
| 5134 | Carers and caregivers |
| 3239 | Nursing associate professionals not elsewhere classified |
| 5133 | Nursing assistants, personal assistants |
| 2225 | Speech therapists |
| 2229 | Health care professionals not elsewhere classified |
| 2491 | Psychologists |
| 1318 | Managers of small enterprises in healthcare and social work |
| 4214 | Pawnbrokers and moneylenders |
| **Women** | |
| 5153 | Prison guards |
| 3234 | Psychiatric nurses |
| 2233 | Emergency room nurses |
| 3233 | Geriatric nurses |
| 3231 | Nursing associate professionals |
| 3235 | Radiology nurses |
| 3239 | Nursing associate professionals not elsewhere classified |
| 3232 | Surgery nurses |
| 2235 | District nurses |
| 5132 | Assistant nurses and hospital ward assistants |
| 3221 | Occupational therapists |
| 2221 | Medical doctors |
| 2492 | Social workers and curators |
| 2491 | Psychologists |
| 3228 | Pharmaceutical assistants |
| 2225 | Speech therapists |
| 2229 | Health care professionals not elsewhere classified |
| 3226 | Physiotherapists and related associate professionals |
| 5133 | Nursing assistants, personal assistants |
| 3461 | Social workers and related associate professionals |

**Table S4. Twenty occupations with the highest exposure to confrontation by sex**

| **Men** | |
| --- | --- |
| Occupational code | Occupational title |
| 5153 | Prison guards |
| 3310 | Pre-primary education teaching associate professionals |
| 3461 | Social workers and related associate professionals |
| 4224 | Transport information clerks |
| 2340 | Special education teaching professionals |
| 5131 | Child-care workers |
| 5111 | Travel attendants and travel stewards |
| 3450 | Police officers and detectives |
| 2330 | Primary education teaching professionals |
| 1318 | Managers of small enterprises in healthcare and social work |
| 5112 | Transport conductors |
| 5134 | Carers and caregivers |
| 3234 | Psychiatric nurses |
| 4214 | Pawnbrokers and moneylenders |
| 4215 | Debt-collectors and related workers |
| 4213 | Croupiers |
| 2323 | Teacher in aesthetic and practical subjects |
| 8340 | Ships' deck crews and related workers |
| 5224 | Kiosk managers |
| 3472 | Radio, television and other announcers |
| **Women** | |
| 5153 | Prison guards |
| 4215 | Debt-collectors and related workers |
| 4214 | Pawnbrokers and moneylenders |
| 2330 | Primary education teaching professionals |
| 2340 | Special education teaching professionals |
| 3234 | Psychiatric nurses |
| 1316 | Managers of small enterprises in public administration |
| 3450 | Police officers and detectives |
| 3310 | Pre-primary education teaching associate professionals |
| 3461 | Social workers and related associate professionals |
| 5112 | Transport conductors |
| 5149 | Other personal services workers not elsewhere classified |
| 9121 | Maids |
| 5134 | Carers and caregivers |
| 3417 | Appraisers, valuers and auctioneers |
| 3152 | Safety, health and quality inspectors |
| 5151 | Firefighters |
| 5152 | Watchmen and security guards |
| 3441 | Customs and border inspectors |
| 2323 | Teacher in aesthetic and practical subjects |

**Table S5. Translated items used for job control and social support at work in the Job Exposure Matrices**

| Job control | Can you partially decide when tasks should be done? |
| --- | --- |
|  | Do you have the opportunity to decide your own work pace? |
|  | Can you take short breaks to talk pretty much any time? |
|  | Are you ever involved in deciding how your work is organised? |
|  | Is there any apprenticeship or introductory training required at the workplace (besides education or course) before you can do your job? |
|  | Does the work require you to repeat the same work steps many times an hour? |
|  | Do you spend quite some time during the day trying to understand or solving difficult problems? |
|  | Does the work offer you the possibility to learn something new and to develop in this occupation? |
| Social support at work | Do you have the opportunity to get support and encouragement from co-workers, when the work feels awkward? |
|  | Do you have the opportunity to get support and encouragement from managers, when the work feels difficult? |
|  | If the tasks feel so difficult, do you then have the opportunity to get advice or help? |
|  | Does it happen that your boss shows appreciation for something you have done? |
|  | Do other people show appreciation for something you have done? |

**Table S6. Hazard ratios (95% CI) for cardiovascular disease, coronary heart disease and stroke by job control and social support at work**

|  | **Cardiovascular disease** | **Coronary heart disease** | **Stroke** |
| --- | --- | --- | --- |
|  | **Model 1**  **HR (95% CI)** | **Model 1**  **HR (95% CI)** | **Model 1**  **HR (95% CI)** |
| **Women** |  |  |  |
| **Job control** |  |  |  |
| High | Ref | Ref | Ref |
| Low | 1.14 (1.11-1.17) | 1.20 (1.16-1.24) | 1.09 (1.06-1.12) |
| **Social support at work** |  |  |  |
| High | Ref | Ref | Ref |
| Low | 1.08 (1.05-1.10) | 1.09 (1.06-1.13) | 1.07 (1.03-1.10) |
| **Men** |  |  |  |
| **Job control** |  |  |  |
| High | Ref | Ref | Ref |
| Low | 1.15 (1.14-1.17) | 1.16 (1.13-1.18) | 1.15 (1.12-1.18) |
| **Social support at work** |  |  |  |
| High | Ref | Ref | Ref |
| Low | 1.01 (0.99-1.02) | 1.00 (0.98-1.02) | 1.01 (0.99-1.04) |

Model 1 adjusting for age, birth year, civil status, birth country, early-life socioeconomic position, education, mental and cardiometabolic health conditions

**Table S7. Baseline characteristics according to levels of general contact with people by sex**

|  | **Men** | | | **Women** | | |
| --- | --- | --- | --- | --- | --- | --- |
| **General contact with people** | **Low**  **(N=346,098)** | **Medium**  **(N=351,012)** | **High**  **(N=314,665)** | **Low**  **(N=360,267)** | **Medium**  **(N=341,916)** | **High**  **(N=350,808)** |
| Characteristics | % | % | % | % | % | % |
| Age |  |  |  |  |  |  |
| 40-49 | 52.7 | 48.8 | 49.4 | 49.6 | 48.4 | 49.1 |
| 50-60 | 47.3 | 51.2 | 50.6 | 50.4 | 51.6 | 50.9 |
| Foreign born | 14.2 | 9.5 | 12.8 | 14.5 | 13.1 | 11.7 |
| Education years |  |  |  |  |  |  |
| ≤9 | 22.9 | 17.9 | 15.2 | 17.5 | 12.0 | 7.1 |
| 10-11 | 38.2 | 36.9 | 29.4 | 35.6 | 38.5 | 33.2 |
| 12 | 12.5 | 14.5 | 14.2 | 16.9 | 12.0 | 11.0 |
| 13-15 | 12.8 | 13.4 | 18.0 | 14.5 | 21.7 | 16.2 |
| >15 | 13.6 | 17.3 | 23.2 | 15.5 | 15.8 | 32.5 |
| Civil status |  |  |  |  |  |  |
| Married/Partnered | 53.5 | 56.4 | 57.4 | 56.5 | 57.4 | 58.2 |
| Unmarried | 32.7 | 29.0 | 26.3 | 24.1 | 22.3 | 21.8 |
| Divorced | 13.2 | 14.0 | 15.7 | 17.6 | 18.4 | 18.3 |
| Widowed | 0.6 | 0.6 | 0.6 | 1.8 | 1.9 | 1.7 |
| Parents’ occupation |  |  |  |  |  |  |
| Non-manual higher level | 3.7 | 5.4 | 7.0 | 5.0 | 4.6 | 5.6 |
| Non-manual intermediate level | 13.9 | 16.7 | 17.3 | 15.7 | 14.4 | 15.8 |
| Non-manual assistant | 8.2 | 10.3 | 12.5 | 9.9 | 10.1 | 10.5 |
| Skilled manual | 24.1 | 25.8 | 21.4 | 23.6 | 24.1 | 23.5 |
| Non-skilled manual | 25.4 | 24.0 | 22.6 | 23.6 | 24.8 | 23.6 |
| Farmer | 9.0 | 6.3 | 5.0 | 6.3 | 7.2 | 7.5 |
| No record | 15.7 | 11.5 | 14.2 | 15.9 | 14.8 | 13.5 |
| History of psychiatric diagnosis | 3.8 | 3.6 | 4.1 | 3.8 | 4.7 | 4.3 |
| History of medication use for hypertension or dyslipidemia | 17.0 | 17.9 | 18.3 | 18.5 | 19.1 | 18.4 |
| History of medication use for diabetes | 3.3 | 3.1 | 3.4 | 1.9 | 2.1 | 1.8 |
| Low job control | 58.9 | 42.9 | 53.5 | 38.8 | 45.5 | 66.3 |
| Low social support | 31.8 | 64.4 | 54.4 | 38.0 | 65.2 | 53.1 |

**Table S8. Baseline characteristics according to levels of emotional demands by sex**

|  | **Men** |  |  | **Women** |  |  |
| --- | --- | --- | --- | --- | --- | --- |
| **Emotional demands** | **Low**  **(N=344,267)** | **Medium**  **(N=335,986)** | **High**  **(N=331,522)** | **Low**  **(N=351,430)** | **Medium**  **(N=359,108)** | **High**  **(N=342,453)** |
| Characteristics | % | % | % | % | % | % |
| Age |  |  |  |  |  |  |
| 40-49 | 52.6 | 51.1 | 47.0 | 51.9 | 47.4 | 47.8 |
| 50-60 | 47.4 | 48.9 | 53.0 | 48.1 | 52.6 | 52.2 |
| Foreign born | 9.6 | 10.8 | 16.2 | 13.0 | 13.0 | 13.3 |
| Education years |  |  |  |  |  |  |
| ≤9 | 18.7 | 22.5 | 15.1 | 18.4 | 11.2 | 7.0 |
| 10-11 | 36.9 | 39.1 | 28.9 | 37.6 | 30.2 | 39.7 |
| 12 | 13.2 | 14.2 | 13.7 | 17.7 | 11.6 | 10.6 |
| 13-15 | 14.8 | 12.1 | 17.1 | 12.2 | 22.7 | 17.2 |
| >15 | 16.4 | 12.1 | 25.2 | 14.1 | 24.3 | 25.5 |
| Civil status |  |  |  |  |  |  |
| Married/Partnered | 56.4 | 54.6 | 56.2 | 56.3 | 59.6 | 56.0 |
| Unmarried | 30.4 | 30.6 | 27.2 | 24.6 | 21.4 | 22.2 |
| Divorced | 12.6 | 14.2 | 16.0 | 17.3 | 17.2 | 19.9 |
| Widowed | 0.6 | 0.6 | 0.6 | 1.8 | 1.8 | 1.9 |
| Parents’ occupation |  |  |  |  |  |  |
| Non-manual higher level | 4.9 | 4.5 | 6.6 | 5.1 | 5.4 | 4.6 |
| Non-manual intermediate level | 16.5 | 14.8 | 16.4 | 15.7 | 16.4 | 13.8 |
| Non-manual assistant | 9.5 | 10.4 | 11.1 | 10.4 | 10.8 | 9.2 |
| Skilled manual | 25.1 | 24.9 | 21.5 | 23.8 | 23.3 | 24.1 |
| Non-skilled manual | 23.9 | 26.3 | 21.9 | 23.9 | 22.9 | 25.2 |
| Farmer | 8.8 | 6.5 | 5.1 | 6.5 | 6.8 | 7.7 |
| No record | 11.3 | 12.6 | 17.4 | 14.6 | 14.4 | 15.4 |
| History of psychiatric diagnosis | 3.0 | 3.8 | 4.7 | 3.7 | 3.9 | 5.2 |
| History of medication use for hypertension or dyslipidemia | 16.5 | 17.7 | 18.9 | 17.7 | 18.2 | 20.1 |
| History of medication use for diabetes | 2.8 | 3.3 | 3.6 | 1.9 | 1.8 | 2.2 |
| Low job control | 41.2 | 51.5 | 62.7 | 39.6 | 46.0 | 65.3 |
| Low social support | 49.1 | 43.0 | 58.5 | 30.6 | 58.3 | 67.0 |

**Table S9. Baseline characteristics according to levels of confrontation by sex**

|  | **Men** |  |  | **Women** |  |  |
| --- | --- | --- | --- | --- | --- | --- |
| **Confrontation** | **Low**  **(N=339,968)** | **Medium**  **(N=338,423)** | **High**  **(N=333,384)** | **Low**  **(N=360,716)** | **Medium**  **(N=352,227)** | **High**  **(N=340,048)** |
| Characteristics | % | % | % | % | % | % |
| Age |  |  |  |  |  |  |
| 40-49 | 51.9 | 50.7 | 48.2 | 50.8 | 48.5 | 47.7 |
| 50-60 | 48.1 | 49.3 | 51.8 | 49.2 | 51.5 | 52.3 |
| Foreign born | 12.0 | 10.2 | 14.3 | 15.3 | 11.7 | 12.3 |
| Education years |  |  |  |  |  |  |
| ≤9 | 21.3 | 21.5 | 13.3 | 17.5 | 10.0 | 9.1 |
| 10-11 | 40.1 | 36.6 | 28.2 | 31.1 | 42.2 | 34.0 |
| 12 | 12.7 | 13.8 | 14.7 | 15.5 | 15.2 | 9.2 |
| 13-15 | 12.4 | 12.3 | 19.3 | 15.1 | 14.6 | 22.7 |
| >15 | 13.5 | 15.8 | 24.5 | 20.8 | 18.0 | 25.0 |
| Civil status |  |  |  |  |  |  |
| Married/Partnered | 53.5 | 56.1 | 57.6 | 57.0 | 57.5 | 57.5 |
| Unmarried | 32.5 | 29.3 | 26.5 | 23.7 | 22.5 | 21.9 |
| Divorced | 13.5 | 14.1 | 15.3 | 17.5 | 18.2 | 18.8 |
| Widowed | 0.5 | 0.6 | 0.6 | 1.8 | 1.8 | 1.8 |
| Parents’ occupation |  |  |  |  |  |  |
| Non-manual higher level | 4.6 | 4.6 | 6.8 | 6.2 | 4.4 | 4.6 |
| Non-manual intermediate level | 14.7 | 15.3 | 17.8 | 16.3 | 14.5 | 15.2 |
| Non-manual assistant | 9.0 | 10.0 | 12.0 | 10.0 | 10.1 | 10.4 |
| Skilled manual | 25.5 | 24.2 | 21.7 | 22.3 | 24.8 | 24.1 |
| Non-skilled manual | 25.3 | 25.6 | 21.2 | 22.2 | 25.3 | 24.4 |
| Farmer | 7.1 | 8.6 | 4.8 | 6.5 | 7.4 | 7.1 |
| No record | 13.8 | 11.7 | 15.7 | 16.5 | 13.5 | 14.2 |
| History of psychiatric diagnosis | 3.8 | 3.5 | 4.1 | 3.8 | 4.2 | 4.8 |
| History of medication use for hypertension or dyslipidemia | 16.9 | 17.6 | 18.5 | 17.8 | 19.3 | 18.9 |
| History of medication use for diabetes | 3.2 | 3.2 | 3.5 | 1.8 | 2.0 | 2.1 |
| Low job control | 61.1 | 44.4 | 49.5 | 46.1 | 60.5 | 43.7 |
| Low social support | 39.5 | 61.4 | 49.6 | 47.9 | 29.7 | 78.9 |
